# From Awareness to Action Study: Improving Human Papillomavirus Knowledge, Screening and Vaccine Uptake Among Mother‐Adolescent Pairs in the HOMINY Study in Nigeria: A Longitudinal Study

**DOI:** 10.1002/jia2.70164

**Published:** 2026-07-24

**Authors:** Oluwaseun Peter, Elohor Oborevwori, Esosa Osagie, Paul Akhigbe, Nosakhare L. Idemudia, Ozoemene Obuekwe, Fidelis E. Eki‐Udoko, Nicholas Schlecht, Yana Bromberg, Praise O. Okoh‐Aihe, Nosayaba Osazuwa‐Peters, Modupe O. Coker, HOMINY Study Group

**Affiliations:** ^1^ Institute of Human Virology Nigeria International Research Centre of Excellence Federal Capital Territory Nigeria; ^2^ Johns Hopkins School of Nursing Johns Hopkins University Baltimore Maryland USA; ^3^ Medical Laboratory Services Department of Medical Microbiology University of Benin Teaching Hospital Benin Nigeria; ^4^ Department of Oral and Maxillofacial Surgery University of Benin Benin Nigeria; ^5^ Department of Child Health University of Benin Teaching Hospital Benin Nigeria; ^6^ Roswell Park Comprehensive Cancer Center Cancer Prevention and Control New York New York USA; ^7^ Department of Biology Emory University Atlanta Georgia USA; ^8^ Department of Basic and Translational Sciences School of Dental Medicine University of Pennsylvania Philadelphia Pennsylvania USA; ^9^ Department of Head and Neck Surgery & Communication Sciences Duke University School of Medicine Durham North Carolina USA; ^10^ Department of Population Health Sciences Duke University School of Medicine Durham North Carolina USA; ^11^ Duke Cancer Institute Durham North Carolina USA; ^12^ Department of Oral Biology School of Dental Medicine Rutgers University Newark New Jersey USA; ^13^ Department of Epidemiology Geisel School of Medicine at Dartmouth Hanover New Hampshire USA

**Keywords:** awareness, cancer, HIV, human papillomavirus, screening, vaccination

## Abstract

**Introduction:**

Persistent cervical infection with high‐risk human papillomavirus (HPV) in women is a leading cause of cervical cancer, and its co‐infection among people living with HIV increases the risk of other HPV‐associated cancers, including oropharyngeal and anogenital cancers. In sub‐Saharan Africa, awareness of HPV is limited, screening and vaccine adoption are critically low, undermining efforts towards sexually transmitted infection (STI) elimination.

**Methods:**

**F**rom **A**wareness to A**CT**ion (FACT) study assessed HPV knowledge, screening and vaccine uptake among adolescent−mother pairs participating in the HOMINY (**H**PV, Human Immunodeficiency Virus, and **O**ral **M**icrobiota **I**nterplay in **N**igerian **Y**ouths) prospective cohort study. Participants were enrolled, including adolescents aged 9–18 years (*N* = 636) and mothers aged 29–59 years (*N* = 385). FACT was conducted at the University of Benin Teaching Hospital, Nigeria, between April 2024 and July 2025, with adolescents and mothers grouped by HIV serostatus. In line with the national immunization programme protecting girls in Nigeria, sensitization programmes were integrated into the research study to promote awareness and adoption of screening and vaccination practices. Knowledge and attitudes regarding HPV and its vaccination benefits were assessed through thematic discussions, interactive sessions and questionnaires administered over the study period.

**Results:**

HPV awareness was initially low, particularly among adolescents, but increased following sensitization, rising from 34.5% to 64.4% among mothers and from 1.4% to 19.0% among adolescents. Vaccination uptake rose from 0% to 5.4% (*p* < 0.0001) in adolescents aged 9–14 years. Among adolescents with perinatally acquired HIV, uptake increased from 1.9% to 4% over time (*p* = 0.003), and the proportion of mothers who underwent HPV‐related screening increased from 38.7% pre‐sensitization to 52.4% 12‐month post‐sensitization (*p* < 0.0001). Barriers to the adoption of preventive services included misconceptions, healthcare provider gaps, myths, misinformation, mistrust, scepticism and limited access.

**Conclusions:**

HPV awareness programmes significantly improved knowledge, vaccination uptake and screening practices in this vulnerable population. As part of comprehensive STI elimination strategies, integrating HPV education and vaccination initiatives into HIV care and research will enhance prevention and address significant barriers. Lessons from a unique programmatic science framework provide critical insights for scaling vaccine delivery, and the design of future vaccine programmes.

## 1 Introduction

Human papillomavirus (HPV) is the most common viral sexually transmitted infection (STI) worldwide and a leading cause of cervical cancer (CC) globally [1, 2]. With over 95% of CC cases attributable to persistent HPV infection, it has been the predominant cause of oropharyngeal squamous cell carcinoma (OPSCC) in high‐income countries, having surpassed tobacco and alcohol as the traditional cause [3, 4]. The World Health Organization (WHO) in 2022 estimated that the majority of sexually active individuals would acquire at least one type of HPV during their lifetime. While most acquired HPVs are short‐lived and cleared spontaneously by the immune system, persistent infection with high‐risk HPV types can lead to serious health conditions, including CC, OPSCC, other anogenital cancers and genital warts [1].

In 2022, Nigeria recorded an estimated 13,676 new CC cases and 7093 deaths, many of which are preventable through HPV vaccination and routine screening [2, 5]. Although HPV poses a risk to the general population, people living with HIV (PLHIV), particularly women and adolescent girls, are disproportionately affected [5, 6]. Their weakened immune systems heighten susceptibility to HPV acquisition and increase the likelihood of progression to malignancy [7]. With effective HPV vaccines available, awareness and uptake in Nigeria remain critically low [1, 4, 8]. Research has consistently revealed substantial knowledge gaps concerning HPV transmission modes, screening, risk factors and vaccine safety within these populations [8, 9, 10].

In 2014, WHO identified the HPV vaccine as a public health priority, recommending that it be included in the national immunization programme. Nigeria, backed by GAVI in 2023, took a cue from this when it introduced and incorporated the single‐dose Gardasil 4‐Valent HPV vaccine, an Immunogen—Recombinant L1 proteins from HPV types 6, 11, 16, 18 [4, 11], into the national routine immunization system for girls aged 9–14 years in October 2023 and May 2024 [10]. This marked a significant milestone in Nigeria's efforts to eliminate CC, as the vaccine is 99% effective when administered [12, 13]. The success of single‐dose HPV vaccination strategies has been demonstrated in recent trials [14, 15]. Therefore, Nigeria's introduction of single‐dose HPV vaccination presents an opportunity to accelerate progress towards the WHO's CC elimination goals [12, 16]. As sub‐Saharan Africa (SSA) bears a disproportionate burden of both HIV and HPV‐related cancers, community‐research partnerships provide valuable models for implementing evidence‐based, contextually appropriate interventions that advance global STI elimination efforts [17].

Mothers and their children living with HIV, as well as the general population, exhibit a heightened vulnerability to HPV acquisition [6, 7, 18]. Therefore, it is crucial to assess their knowledge of HPV, screening practices and vaccine uptake, while also identifying barriers to vaccination to guide targeted public health interventions [15, 19]. In low‐ and middle‐income countries (LMICs), where both HIV and HPV co‐infection prevalence is not fully known and where the study is focused, integrating HPV education and immunization strategies into HIV care and broader public awareness efforts could play a vital role in reducing the burden of CC and related diseases [20, 21].

**F**rom **A**wareness to A**CT**ion (FACT) study was developed within the parent prospective cohort study in Nigeria, HOMINY (**H**PV, Human Immunodeficiency Virus, and **O**ral **M**icrobiota **I**nterplay in **N**igerian **Y**ouths) study, which investigates the impact of the microbiome on the increased susceptibility to HPV acquisition [18, 22, 23]. Building on the HOMINY study, the FACT study seeks to assess HPV‐related knowledge, including awareness of transmission modes, CC screening, and HPV vaccination among adolescents and mothers enrolled in the cohort. In addition, the study evaluates whether a structured sensitization programme based on training, education and awareness improves participants’ knowledge and awareness of HPV. The study further aims to longitudinally assess the impact of this programme on the uptake of HPV‐related preventive services, including CC screening and HPV vaccination, within the context of the ongoing national HPV vaccine implementation programme in Nigeria [24].

## 2 Methods

### 2.1 Study Participants

The FACT study implementation began at the second study visit of the parent study, HOMINY, which was a longitudinal HIV cohort study with a unique mother−adolescents population of PLHIV and HIV‐negative individuals, conducted at the University of Benin Teaching Hospital (UBTH), Benin City, Nigeria [18, 22]. Participants were recruited from the paediatric special treatment HIV clinic for adolescents with perinatally acquired HIV (HI) on Antiretroviral Therapy (ART) and HIV‐exposed without acquisition (HEU) adolescents born to mothers living with HIV (HI), as well as from the general practice clinic for adolescents who are HIV‐unexposed (HUU) and their mothers (HU). FACT participants were grouped into 385 mothers aged 29–59 years (262 HI and 123 HU) and 636 adolescents aged 9–18 years (220 HI, 203 HEU and 213 HU), totalling 1021. Participants were enrolled in April 2024, pre‐sensitization data were collected and they were followed through three visits at 6‐month intervals, from sensitization to 1‐year post‐sensitization ending July 2025 [22].

### 2.2 Data Collection Procedure

This study employed a mixed‐methods design, with quantitative and qualitative data guided by the Kirkpatrick model [25], to evaluate the effectiveness of a research implementation and training programme.

### 2.3 Quantitative Data

Data collection was conducted longitudinally across three of the four scheduled clinical visits. A standardized and structured questionnaire was adapted and administered at each visit to comprehensively capture data relevant to HPV, with a particular emphasis on HPV screening, vaccine awareness and uptake [18, 26, 27]. Questionnaire items were informed by well‐established HPV knowledge and awareness constructs described by Waller et al. [28]. The adaptation of existing instruments for the Nigerian context is an established and accepted approach, as demonstrated in previous studies conducted in similar settings [21]. Based on a review of the literature, screening items were constructed, covering key domains including sexual health history, HPV vaccination status, symptom presentation, and risk factor assessment. Detailed socio‐demographic information was collected through validated interviewer‐administered questionnaires from both adolescents and their mothers or guardians [22]. Variables assessed included age, sex, education level, income, and employment status. These factors were collected to evaluate their association with HPV exposure, health‐seeking behaviour and access to care [18, 22]. Sexual health data were obtained using a confidential, age‐appropriate questionnaire administered in a private setting by trained personnel. These data were critical for evaluating potential transmission pathways, particularly in relation to HPV. Medical history, including screening for HPV such as visual inspection with acetic acid (VIA) and papanicolaou smear (PAP), viral load for PLHIV, and vaccination status were documented using a standardized protocol [22].

This mixed‐methods approach combining clinical examination, medical record review and structured interviews enabled a robust assessment of the complex interplay between social determinants, sexual behaviours and infectious disease risk in the mother−adolescents population.

### 2.4 Qualitative Data

Qualitative data were collected during sensitization sessions provided to FACT study participants over a period of 3 weeks through thematic discussions and interactive sessions, with 60 mother−adolescent pairs per week as a subset of the entire population. Adolescents paired with their mothers were selected through criterion‐based purposive sampling to ensure that all participants met the predefined age specifications for vaccination [29]. Nine trained study personnel with a combined experience of over 15 years coordinated each session, maintaining a participant‐to‐facilitator ratio of 7 to 1.

The programme evaluation was guided by the Kirkpatrick model, which assesses training and educational interventions across four hierarchical levels: reaction, learning, behaviour and results, offering a comprehensive framework for integrating the qualitative and quantitative components of the study and for understanding the programme's impact [25].

The four sensitization sessions addressed the first two levels of the model, reaction and learning [25]. Reaction was evaluated through participants’ responses regarding prior awareness of HPV acquisition, CC screening, HPV vaccination services and associated costs. Learning was assessed through guided qualitative discussions and questions exploring participants’ knowledge and perceptions of HPV acquisition and CC using a structured discussion guide (File S1). Education and sensitization were subsequently provided on each of these during the interactive sessions to improve participants’ knowledge and awareness of HPV acquisition, transmission modes, screening and vaccination. Post‐session tests were conducted using similar questions to evaluate knowledge acquisition.

The third and fourth level of the Kirkpatrick model, behaviour and results, were evaluated longitudinally through participants’ uptake of CC screening and HPV vaccination during follow‐up visits [22, 25]. Behaviour was assessed through self‐reported screening and vaccination, while results were measured through observed changes in HPV awareness, screening and vaccine uptake over time.

We documented participants’ responses, reactions and learning experiences through field notes recorded in English. These were reviewed and organized by the research team using thematic analysis. Responses relating to participants’ reactions, knowledge acquisition, perceived barriers and attitudes towards HPV screening and vaccination were coded and grouped into recurring themes. Discrepancies in interpretation were resolved through team discussion to ensure consistency in coding and theme development to ensure consistency and completeness.

### 2.5 Intervention

In brief, the sensitization programme was conducted in English. It comprised four sessions, each lasting approximately 15–20 min. We assessed the participants’ previous knowledge of HPV; trained them on HPV transmission modes, screening and vaccination benefits; held interactive sessions to address questions from participants and clarify misconceptions.

The intervention was implemented over a 3‐week period among mother−adolescent pairs enrolled in the FACT study.

Participants were encouraged to undergo screening within the facility where the research was ongoing with support from the research team, and to take advantage of the upcoming free vaccination programme organized by the government. Additionally, questionnaires administered at every visit (e.g. “Since the last visit, have you had an HPV test?” for mothers and “Since the last visit, have you had HPV vaccine?” for adolescents) served as prompts for participants.

### 2.6 Ethics

The ethical considerations for the study design were reviewed and approved by the institutional review boards at the Rutgers State University of New Jersey (Pro2022000949) and the University of Benin Teaching Hospital, Benin City (ADM/E22/A/VOL. VII/14813674), Nigeria. Informed consent and assent were obtained from all participants.

### 2.7 Data Analysis

Study data were collected and managed using REDCap electronic data capture tools hosted by the University of Pennsylvania [30]. All analyses were conducted using R version 4.5.0, R Foundation for Statistical Computing, Vienna, Austria [31]. Pre‐sensitization characteristics were summarized by study groups separately for mothers and adolescents using mean ± standard deviation (SD) for continuous variables and frequencies with percentages for categorical variables.

HPV‐related awareness and knowledge were assessed as distinct constructs. Awareness referred to familiarity with HPV or HPV vaccination (e.g. having heard of HPV or the HPV vaccine). Knowledge referred to understanding of HPV‐related concepts, including transmission, disease association and prevention (e.g. sexual transmission, transmission through kissing, association with CC and vaccine prevention). For regression analyses, a binary HPV knowledge variable was defined based on responses to whether HPV could be transmitted sexually and/or through kissing; participants responding “Yes” to either item were classified as having HPV knowledge, while those responding “No” to both items were classified as not having HPV knowledge.

Longitudinal analyses included repeated observations across pre‐sensitization, 6‐month post‐sensitization and 1‐year post‐sensitization, respectively. Associations between HPV knowledge and sexual practices (mouth kissing, oral sex, vaginal sex, anal sex and condom use among sexually active participants) were estimated using Poisson regression with a log link to obtain prevalence ratios (PRs) and 95% confidence intervals (CIs). Repeated measures across visits were accounted for using cluster‐robust standard errors at the participant level. Adjusted models included visit, age and education level. Trends in vaccination and screening outcomes across visits were assessed using Cochran−Armitage trend tests. Missing data were handled using complete‐case analysis, with observations missing variables required for a given analysis excluded from that model. Statistical significance was defined as two‐sided *p* < 0.05.

## 3 Results

### 3.1 Quantitative Results

#### 3.1.1 Participant Socio‐Demographic and Pre‐Sensitization Characteristics

As summarized in Tables 1 and 2, a total of 636 age‐ and sex‐matched adolescents (mean age 13.4 ± 2.5 years; range 9–18 years) and 385 mostly middle‐aged mothers were enrolled. Adolescents’ sex distribution and the ages of mother−adolescent pairs are presented descriptively by study group. Most participants had at least secondary education; 29.0% of mothers had tertiary education, while more than 50% of adolescents were still in secondary school. Among mothers living with HIV, 82.5% had a viral load <20 copies/mL, compared with 72.9% of Adolescents living with HIV (ALHIV).

**TABLE 1:** Socio‐demographic characteristics of adolescents.

	HI	HEU	HUU	
Characteristics	*N* = 220^a^	*N* = 203^a^	*N* = 213^a^	*p*‐value^b^
Age (years)				0.001
Mean ± SD	13.7 ± 2.5	12.9 ± 2.3	13.6 ± 2.5	
Gender				>0.9
Female	113 (51.4%)	108 (53.2%)	113 (53.1%)	
Male	107 (48.6%)	95 (46.8%)	100 (46.9%)	
Education				0.009
Primary	84 (42.2%)	75 (39.1%)	60 (31.7%)	
Secondary	114 (57.3%)	112 (58.3%)	123 (65.1%)	
Tertiary	1 (0.5%)	2 (1.0%)	6 (3.2%)	
Others	0 (0.0%)	3 (1.6%)	0 (0.0%)	
School attended				
Public	110 (50.0%)	118 (58.2%)	106 (49.8%)	
Private	51 (23.2%)	48 (23.6%)	43 (20.2%)	
NA	59 (26.8%)	37 (18.2%)	64 (30.0%)	
Viral load scale				>0.9
<20 copies/mL	159 (72.9%)	0 (NA%)	0 (NA%)	
20+ copies/mL	59 (27.1%)	0 (NA%)	0 (NA%)	

Education: Adolescent data used were collected at 12‐month post‐sensitization. Percentages were calculated using non‐missing observations within each study group: HI (*n* = 199), HEU (*n* = 192), and HUU (*n* = 189).

Abbreviations: HEU, adolescents HIV‐exposed without acquisition; HI, adolescents living with HIV; HUU, adolescents HIV‐unexposed; SD, standard deviation; Study group codes.

*p*‐values: Analysis of variance (ANOVA) for continuous variables in adolescents; Fisher's exact test for categorical variables.

^a^ *n* (%).

^b^ One‐way analysis of means; Fisher's exact test.

**TABLE 2:** Socio‐demographic characteristics of mothers.

	HI	HU	
Characteristics	*N* = 262^a^	*N* = 123^a^	*p*‐value^b^
Age (years)			0.052
Mean ± SD	44.2 ± 5.1	43.1 ± 5.6	
Education			<0.001
Primary	72 (27.5%)	14 (11.4%)	
Secondary	130 (49.6%)	60 (48.8%)	
Tertiary	52 (19.8%)	47 (38.2%)	
Others	8 (3.1%)	2 (1.6%)	
Income (Naira)			>0.9
Mean ± SD	47,576.3 ± 67,655.1	47,965 ± 45,413.9	
Viral load scale			>0.9
<20 copies/mL	216 (82.4%)	1 (100.0%)	
20+ copies/mL	46 (17.6%)	0 (0.0%)	

Abbreviations: HI, mothers living with HIV; HU, HIV‐negative mothers; SD, standard deviation; Study group codes.

*p*‐values: *t*‐test for continuous variables in mothers; Fisher's exact test for categorical variables.

^a^ *n* (%).

^b^ Welch two‐sample *t*‐test; Fisher's exact test.

#### 3.1.2 HPV Knowledge and Vaccine Awareness Among Participants

In Table 3, awareness of HPV and its vaccine rose steadily across visits for both groups. Mothers’ HPV knowledge nearly doubled with an increase from 34.5% to 64.4% 1‐year post‐sensitization, and HPV vaccine awareness from 10.9% to 27.6% (*p* < 0.0001 and *p* = 0.023, respectively). Adolescents’ HPV knowledge improved from 1.4% to 19% and vaccine awareness from 0.3% to 12.8%. In absolute terms, mothers showed larger gains (34.5%−64.4%), while adolescents showed larger proportional increases (1.4%−19.0%) from a much lower baseline, predominantly in female adolescents (File S2).

**TABLE 3:** HPV knowledge and vaccine awareness.

Respondent	Visit	*N*	HPV knowledge (%)^a^	HPV vaccine awareness (%)^b^	*p*‐value trend (HPV knowledge)	*p*‐value trend (vaccine awareness)
Mothers	Pre‐sensitization	385	133 (34.5)	42 (10.9)	<0.0001	0.023
Mothers	6‐month visit	359	176 (49.0)	66 (18.4)	<0.0001	0.023
Mothers	1‐year visit	340	219 (64.4)	94 (27.6)	<0.0001	0.023
Youths	Pre‐sensitization	636	9 (1.4)	2 (0.3)	<0.0001	0.392
Youths	6‐month visit	597	52 (8.7)	43 (7.2)	<0.0001	0.392
Youths	1‐year visit	580	110 (19.0)	74 (12.8)	<0.0001	0.392

*p*‐value trends were estimated using Cochran–Armitage trend tests across visits.

^a^ HPV knowledge was defined as responding “Yes” to either of the following: HPV is sexually transmitted and/or HPV can be transmitted through kissing.

^b^ HPV vaccine awareness was defined as self‐reported familiarity with HPV vaccination (“Have you heard of HPV vaccine?”).

#### 3.1.3 Changes in HPV Awareness Following Sensitization

**FIGURE 1:**
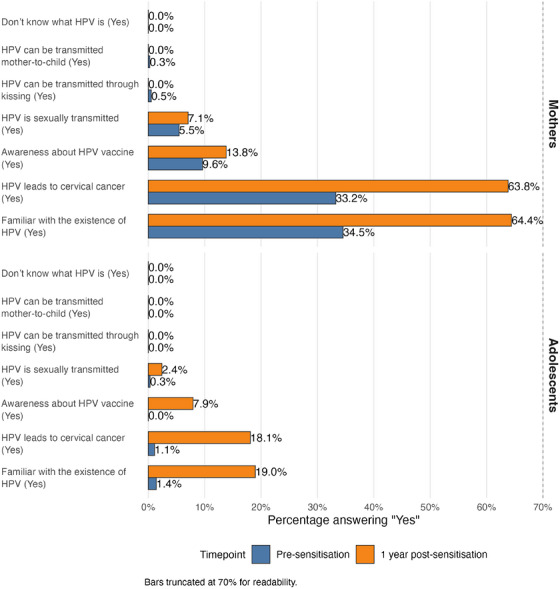
Awareness level about Human Papillomavirus.

As shown in Figure 1, specific knowledge about the transmission modes of HPV was significantly low, with only 0.3% and 0.5% of mothers reporting that HPV can be transmitted vertically and through kissing, respectively. No adolescents responded positively to being aware of either mode of transmission. However, this was not the case for sexual transmission, where a steady increase in awareness was observed across visits. The most significant improvement was seen in knowledge about HPV being causally linked to cancer, with awareness among mothers rising from 33.2% to 63.8%, and among adolescents from 1.1% to 18.1%. Stratified analyses by gender showed greater improvements in HPV knowledge and vaccine awareness among female adolescents than among males. HPV vaccine awareness increased from 0.6% pre‐sensitization to 20.4% 1‐year post‐sensitization among females, compared with 0.0%−4.1% among males. Similarly, HPV knowledge increased from 1.5% to 26.2% among females, compared with 0.7%−8.9% among males (File S2).

**FIGURE 2:**
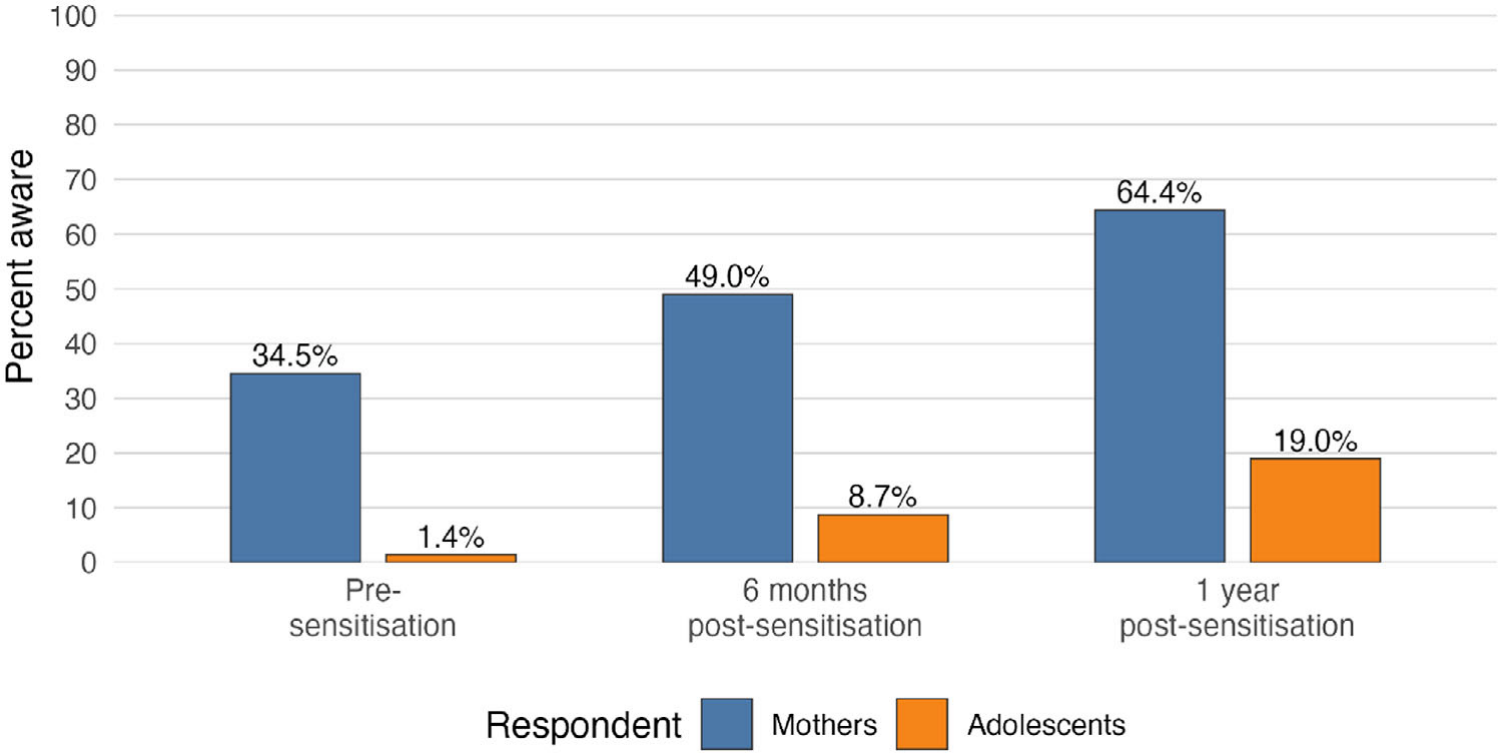
Human Papillomavirus awareness in mother−adolescent dyads across visits.

A positive upward trend in HPV knowledge across visits for both groups was observed in Figure 2, with mothers consistently higher, highlighting the mothers’ stronger retention and comprehension in this older and more experienced group (i.e. mothers vs. adolescents).

#### 3.1.4 Longitudinal Uptake of CC Screening Among Mothers

As shown in Table 4, VIA screening rose steadily from 35.6% pre‐sensitization to 49.7% at final visit (< 0.0001), indicating a highly significant upward trend. The PAP test results remained low and stable (∼ 10%–11%) with a non‐significant *p*‐value of 0.649. There was an increase in any screening (VIA or PAP) from 38.7% to 52.4%, (*p* < 0.0001). Overall, this showed a marked and statistically significant increase in cervical screening, driven almost entirely by the growth in VIA participation.

**TABLE 4:** Screening across visits (mothers only)—with trend *p*‐values.

Visit	*N*	VIA (%)	PAP (%)	Any screening (%)	VIA *p*‐values	PAP *p*‐values	Any screening *p*‐values
Pre‐sensitization	385	137 (35.6)	39 (10.1)	149 (38.7)	<0.0001	0.649	<0.0001
6‐month visit	359	150 (41.8)	38 (10.6)	162 (45.1)	<0.0001	0.649	<0.0001
1‐year visit	340	169 (49.7)	38 (11.2)	178 (52.4)	<0.0001	0.649	<0.0001

Abbreviations: PAP, papanicolaou test; VIA, visual inspection with acetic acid.

**FIGURE 3:**
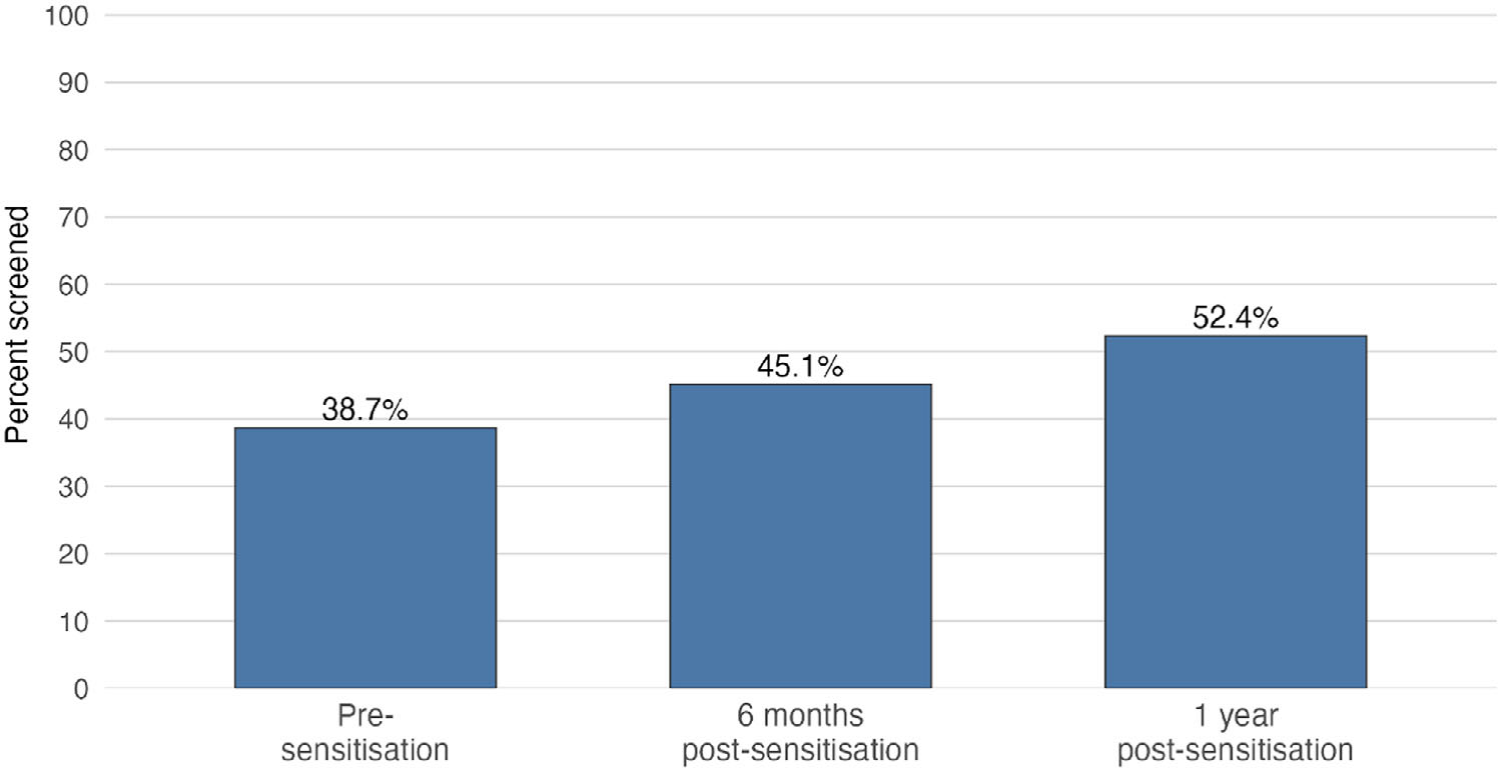
Any cervical screening (VIA or PAP) across visits in mothers. Abbreviations: PAP, papanicolaou test; VIA, visual inspection with acetic acid.

VIA and/or PAP cervical screening tests done by mothers across the visits are illustrated in Figure 3. There was a consistent upward trend in screening for CC in the mothers, post‐sensitization.

#### 3.1.5 Longitudinal HPV Vaccine Uptake Among Adolescents

Table 5 presents the vaccine uptake in adolescents stratified by age group. Prior to sensitization and national vaccine rollout, uptake was 0% in both age groups. Among adolescents aged 9–14 years, uptake increased from 0% at pre‐sensitization to 3.2% at the 6‐month visit and 5.4% at the 1‐year visit (*p*‐trend <0.001). Among adolescents aged 15–18 years, uptake remained low, increasing from 0% at pre‐sensitization to 0.5% and 0.9% at the 6‐month and 1‐year visits, respectively (*p*‐trend = 0.199). Upon stratification by gender and HIV acquisition, vaccine uptake among females increased from 0% to 6.5% (*p*‐trend <0.001), whereas vaccine uptake remained 0.0% among males. Significant changes over time were observed in HI and HEU adolescents (*p* = 0.003 and *p* < 0.001), whereas uptake remained low among HUU adolescents (*p* = 0.186) as shown in File S3.

**TABLE 5:** Vaccine uptake across visits (adolescents only)—with trend *p*‐values.

Age group	Visit	*N*	HPV vaccine dose 1 (%)	Dose *p*‐values
9–14 years	Pre‐sensitization	433	0 (0)	<0.0001
9–14 years	6‐month visit	376	12 (3.2%)	<0.0001
9–14 years	1‐year visit	315	17 (5.4%)	<0.0001
15–18 years	Pre‐sensitization	182	0 (0%)	0.199
15–18 years	6‐month visit	188	1 (0.5%)	0.199
15–18 years	1‐year visit	216	2 (0.9%)	0.199

**FIGURE 4:**
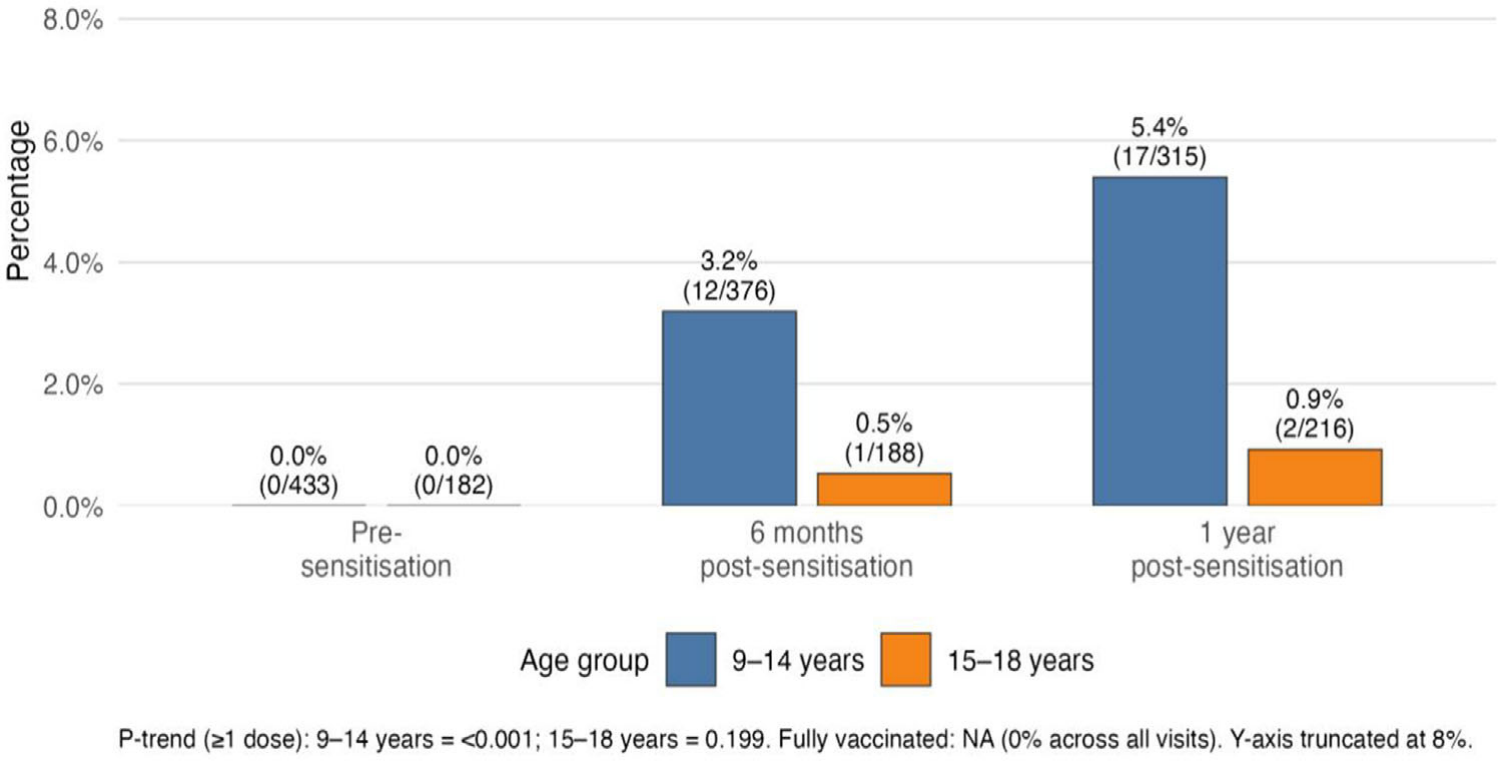
Human Papillomavirus vaccine uptake in adolescents pre‐sensitization and in 6‐month and 1‐year post‐sensitization, respectively.

The vaccine uptake trend in adolescents, depicted in Figure 4, shows a steady rise in vaccine uptake across visits, indicating the impact of the national free vaccination scheme and sensitization.

#### 3.1.6 Barriers to CC Screening Among Mothers

Table 6 shows that the main barrier to screening among mothers was a lack of perceived need when asymptomatic, reported by 60.5% for VIA and 71.7% for PAP testing. Another notable barrier was the absence of a healthcare provider's recommendation, while smaller proportions cited misconceptions or limited knowledge.

**TABLE 6:** Barriers to cervical cancer screening among mothers (counts and percentage).

Barrier	*n*	VIA %	*n*	PAP %
Haven't had any problems	150	60.5	248	71.7
Doctor didn't order it/didn't say I needed it	59	23.8	67	19.4
Put it off/didn't get around to it	15	6.0	4	1.2
Didn't need it/didn't know I needed this type of test	4	1.6	5	1.4
Had hysterectomy	2	0.8	2	0.6
Don't know	18	7.3	20	5.8

Abbreviations: PAP, papanicolaou test; VIA, visual inspection with acetic.

#### 3.1.7 Association Between HPV Knowledge and Sexual Practices

**FIGURE 5:**
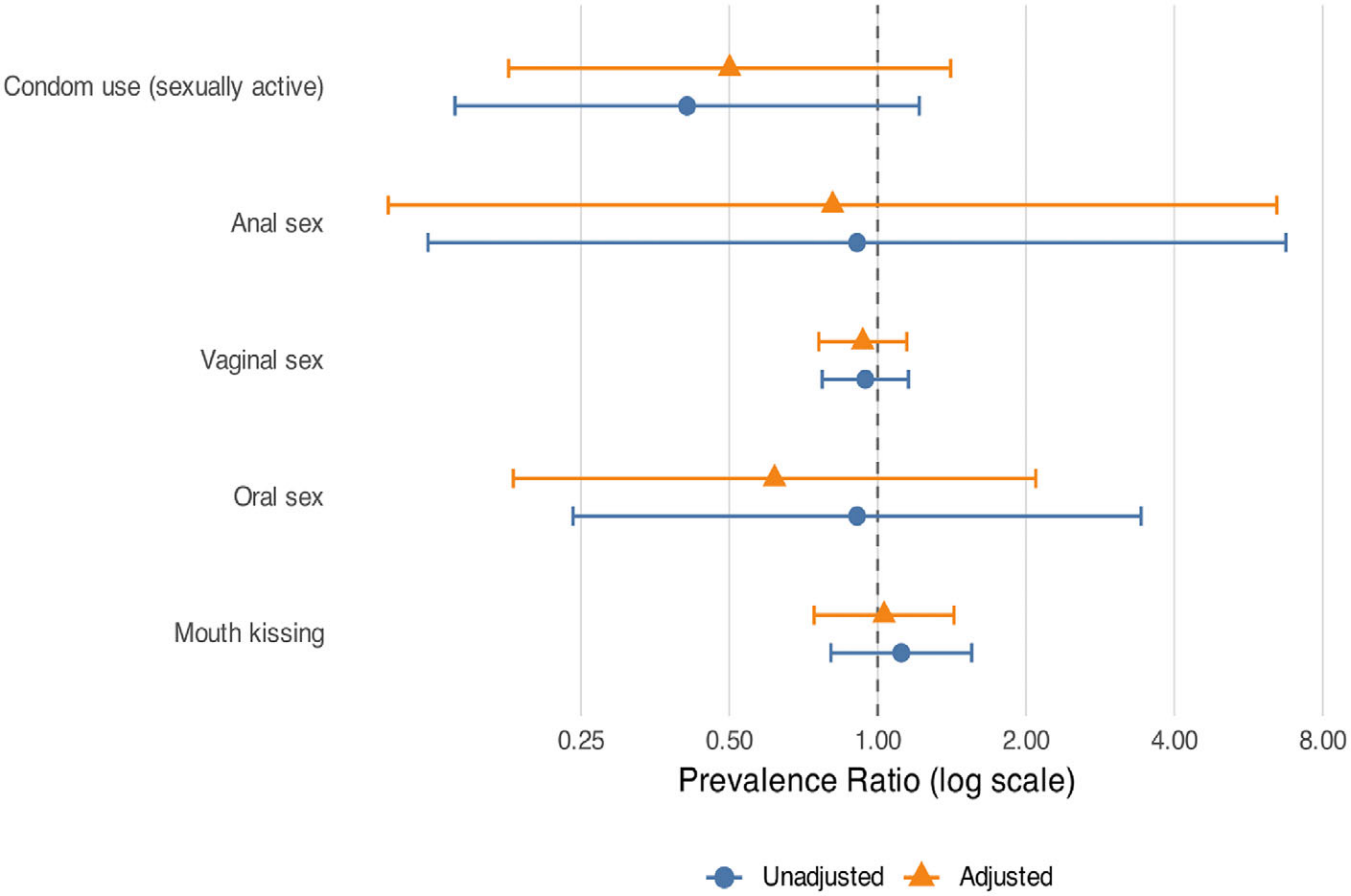
Human Papillomavirus knowledge versus sexual practices (mothers; pre‐sensitization—1‐year post‐sensitization). Prevalence ratios (Poisson log) with cluster‐robust SEs; unadjusted and adjusted for visit, age and education.

Association between HPV knowledge and sexual practices among mothers from the pre‐sensitization to post‐sensitization as shown in Figure 5. Prevalence ratios with 95% confidence intervals (CIs) from Poisson regression models are shown on a logarithmic scale. Both unadjusted and adjusted estimates (for visit, age, and education) clustered around unity, with CIs crossing the null line, indicating no significant association between HPV knowledge and reported sexual practices.

**FIGURE 6:**
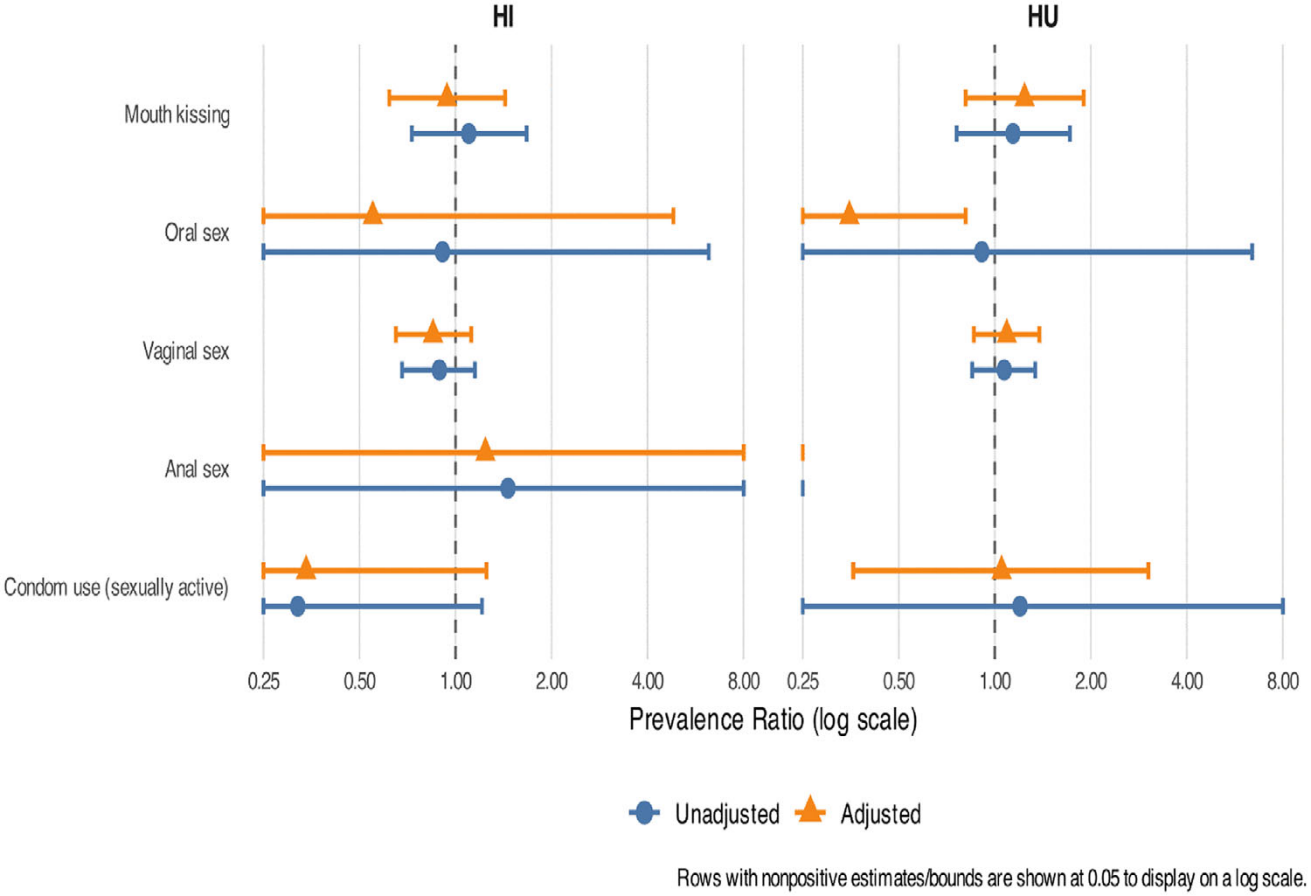
Human Papillomavirus knowledge versus sexual practices (mothers; pre‐sensitization—1‐year post‐sensitization). Subgroup forest plot by study group (HI, HU). Prevalence ratios (Poisson log) with cluster‐robust SEs; unadjusted and adjusted for visit, age and education; Study group codes: HI, mothers living with HIV; HU, HIV‐negative mothers.

Figure 6 shows the association between HPV knowledge and sexual practices among mothers, stratified by HIV serostatus across the study period. Both unadjusted and adjusted prevalence ratios clustered around 1, with confidence intervals crossing the null, indicating no significant association.

### 3.2 Qualitative Results

The 180 mother−adolescent pairs (≈18%; 60 per week) selected for the qualitative study from the total study population of 1021 share the same socio‐demographic characteristics as presented in File S4.

All qualitative quotes presented below are from mothers only, as most adolescents were hearing about HPV for the first time during the sensitization sessions and had limited pre‐sensitization awareness from which to draw for the qualitative discussions.

#### 3.2.1 Misconceptions

One of the commonly cited explanations for the failure to be screened in mothers was that awareness is relatively high, but knowledge of its importance is low, even though VIA is available free of charge in the facility. Some are unaware that screening is free, while others are not aware that the vaccine is more effective in adolescents aged 9–14 years.

“… I did not know the test was free in UBTH, but I don't know why I should do the test since I do not have any health challenge.”

“Can I take the vaccine or is it just to protect my daughter?”

“…I can't have cancer, never! It's not in our family; we don't inherit cancer…”

Some do not even have the knowledge, nor are they aware of any such thing as CC, HPV being the cause, or that it can be prevented by vaccination.

“… I don't know about HPV, I am only aware of HIV. What is this disease [HPV] again?”

#### 3.2.2 Vaccine Mistrust

Some of the FACT study participants were aware of the vaccination; however, they believed that their children did not need it, and others were afraid of potential side effects, especially because it mainly targeted females. Parents/guardians did not think that boys were also at risk of acquiring HPV and were eligible for the vaccination. However, the national programme did not include boys in the vaccination scheme. The various conspiracy theories surrounding COVID‐19 vaccination also played a significant role in shaping attitudes towards the vaccine uptake.

“This vaccine seems to be targeted at Nigeria to reduce our population. I cannot allow my girls to take it…”

“…why are they vaccinating our girl child? They don't want them to get pregnant in the future… I don't want trouble for my child after she gets married and she is not able to give birth… over my dead body.”

“I hope this one [vaccine] won't be worse than COVID 19 vaccine, I could not lift my hand properly after I took the vaccine.”

#### 3.2.3 Healthcare Providers’ Gaps

Another common observation was a lack of knowledge that HPV is responsible for cervical, oropharyngeal, and/or anogenital cancers and transmission modes of HPV, despite some mothers having undergone screening before the implementation of the FACT study. They believed that they were not at risk of CC and did not even know how it could be contracted.

“We were told during our clinic visit that it was now part of the test we need to do because we're living with HIV, but we did not know why or what the cause was.”

“…the doctor just pointed to one of the rooms where the nurses were. He said I should do one test [VIA] that would require them to put something inside my private part to check if I had the infection [cervical cancer] … I don't know what causes it, but after the test, I was told I don't have it [cancer], that I was negative.”

“How can we protect or prevent ourselves from this infection? Maybe we can use protection [condom]…?”

Most of the mothers who underwent screening were PLHIV, with only a few knowing the purpose of the test they were told to do in their clinic.

“I was told it is caused by a virus and I even did what is called Pap smear to be sure I did not have it [cervical cancer] …”

#### 3.2.4 Limited Access

Some participants were interested in undergoing the test and wanted their children to take the vaccine once rollout begins, with concerns about location and cost implications of the screening and vaccination.

“Please, when will they start giving the vaccine and I can instruct my child to be vaccinated? How much will it cost or it free?”

“Where in UBTH will I be able to do the screening, or can we do it in any hospital or primary health centre close to us and how much is it?”

“For the test [VIA], how many days before will it take for the results to be ready, and can my child be tested?”

## 4 Discussion

This study highlights the persistent gap in awareness of HPV, its vaccine uptake and screening in Nigeria, while demonstrating that a targeted low‐cost sensitization programme embedded with a research study can drive some measurable improvements in HPV awareness, CC screening and vaccine uptake in a resource‐limited setting. Pre‐sensitization, HPV knowledge was critically low, reflecting the entrenched knowledge deficit that has been documented across SSA despite decades of global HPV prevention advocacy [32]. By the 12‐month visit, mothers’ HPV knowledge had nearly doubled, VIA screening uptake had risen among women and vaccine uptake among eligible adolescents aged 9–14 years had increased. These gains coincided with Nigeria's national HPV vaccine rollout but were sustained progressively, suggesting an intervention effect beyond the 5‐day national campaign alone. These findings are particularly significant in the context of Nigeria's CC burden; the convergence of a national immunization programme with community‐embedded research platforms represents a critical and time‐sensitive window for promoting awareness. This present study adds to a growing body of evidence by being among the first to longitudinally track HPV awareness and action within mother−adolescent dyads in a context of a research study, using a mixed‐methods design that captures not only whether outcomes changed, but why barriers persisted for those who did not.

Pre‐sensitization, only one‐third of mothers and less than 2% of adolescents had heard of HPV, and vaccine awareness was even lower (10.9% of mothers; 0.3% of adolescents). Similarly, in 2020, HPV knowledge and vaccine awareness were recorded as < 3% and 0.5%, respectively, in secondary school girls by Ezeanochie and Olasimbo [26]. Our findings align with prior regional studies in SSA that describe similarly low baseline knowledge of HPV and the vaccine among both adults and adolescents [8, 9].

Following the structured sensitization aimed at stimulating awareness and action for both screening and vaccine uptake among mothers and adolescents, HPV awareness among mothers increased, and vaccine awareness more than doubled. CC screening showed a similar pattern, while VIA uptake rose from 35.6% pre‐sensitization to 49.7% by the 12‐month visit (*p* < 0.0001), whereas PAP smear rates remained static at ∼10%. This sustained growth in VIA update and stable PAP testing aligns with WHO recommendations that prioritize VIA as a pragmatic first‐line strategy in resource‐limited settings and reflects the greater availability and immediate results advantage of VIA over cytology [33]. Nevertheless, overall screening coverage remains sub‐optimal given Nigeria's high CC burden and falls short of WHO's 70% target, underscoring the continued burden of CC in Nigeria [2, 5].

Adolescent awareness also improved, albeit from a very low base, reaching 19% for HPV and 12.8% for the vaccine by the final visit. Vaccine uptake in adolescents aged 9–14 years rose significantly from 0% pre‐sensitization to 5.4% by the 12‐month visit (*p* < 0.0001). However, despite this improvement, the increase in uptake, although meaningful within the context of a research‐embedded awareness programme, fell far short of the coverage levels needed to achieve population‐level impact and the WHO target of 90% HPV vaccination coverage among girls by age 15 years [16]. In contrast, uptake among adolescents aged 15–18 years remained very low (0.9%), consistent with their exclusion from the national programme, and did not reach statistical significance (*p* = 0.199). The timing and magnitude of these gains seen in 9–14 year‐olds suggest that repeated community engagement and education were key drivers, consistent with evidence that structured outreach and school‐ or clinic‐based education significantly improve HPV knowledge, screening and vaccine acceptance in SSA [1, 13].

Barriers to CC screening include lack of symptoms and absence of recommendation from a provider (∼20%). Our quantitative findings were echoed in qualitative sessions, where provider endorsement emerged as a prominent theme: women who had been directed to screening often did not understand why it was recommended or what the test entailed. This aligns with the strongest predictors of screening in LMICs [34] and underscores missed opportunities during routine clinical encounters [15, 19] with immediate policy relevance as Nigeria scales up its national HPV vaccination [10].

The absence of a significant association between HPV knowledge and sexual practices among mothers, even after further stratification by HIV status, aligns with previous studies suggesting that knowledge acquisition alone does not necessarily translate into behavioural change without addressing underlying attitudes, perceived risk and structural enablers [35]. While educational interventions improve understanding of HPV transmission and prevention, its short‐term impact on modifying intimate behaviours may be limited without accompanying strategies that address barriers to safer practices, long‐standing social norms, relationship dynamics and perceived vulnerability that education alone cannot rapidly alter [36]. This finding is not unique to the Nigerian context, as similar disconnects between HPV knowledge gains and behaviour change have been reported across diverse LMICs [20]. Our findings, therefore, support the integration of behavioural and psychosocial components such as risk perception, social norm reframing and psychosocial supportive frameworks within HPV education programmes targeted at mothers and other adult populations.

Qualitatively, participants’ responses revealed a gap between general awareness and specific actionable knowledge. As an example, participants who had heard of HPV frequently did not know that it causes CC and that the vaccine is most effective at ages 9–14, or that screening can help early detection and prevent spread [1]. This pattern of reasonable surface awareness but poor depth of knowledge that limits preventive action has been reported elsewhere in Nigeria and the region. Studies have documented low detailed knowledge of HPV, CC and prevention despite some awareness campaigns, and link poor knowledge to lower screening and vaccine uptake [37, 38]. Such findings suggest that simple exposure to HPV messaging is insufficient; and programmes must pursue targeted, iterative education that builds from basic awareness towards practical, actionable knowledge of prevention pathways.

Three qualitative themes emerged as prominent barriers: misconceptions about susceptibility, vaccine mistrust and poor provider communication. Many mothers expressed beliefs that they or their family are not susceptible to CC or that screening is unnecessary in the absence of symptoms. Such misconceptions, including the idea that CC is not relevant unless symptoms are present, reduce preventive screening behaviour. Similar findings in Nigeria [39] and SSA [40] have shown that low perceived susceptibility and erroneous beliefs undermine uptake of screening and vaccination. Vaccine hesitancy was shaped by fears about the vaccine's potential effects on fertility, population control conspiracy theories, and memories of adverse events from prior vaccine drives. These themes mirror wider findings from LMICs about vaccine mistrust, amplified by social media and pandemic‐era (such as COVID‐19) conspiracy narratives, which contribute to hesitancy for HPV vaccination, where parents may fear infertility or hidden agendas [27, 36]. Systematic and qualitative reviews link conspiracy beliefs and mistrust to reduced vaccine confidence [15, 41]. Addressing these concerns will require culturally sensitive, community‐led communication that explicitly engages trusted local voices to reframe the vaccine as protective rather than threatening.

Poor provider communication and missed opportunities for education are common barriers identified; healthcare practitioners sometimes fail to explain the link between HPV and cancer or to counsel on prevention, leaving health seekers uncertain about the purpose and benefit of screening/vaccination [36, 40]. Studies from Nigeria show that logistical barriers, such as uncertainty about where services are offered, perceived or real costs, and convenience, meaningfully reduce uptake and must be addressed alongside information campaigns [39, 40]. Even among women who expressed genuine interest in screening or vaccination, practical questions about service location, cost, wait times, and eligibility for themselves versus their daughters remained unanswered. These logistical barriers, seen as meaningful contributors to low uptake in Nigeria, must be addressed alongside knowledge‐building campaigns. Integrating clear, standardized counselling into every clinical encounter, supported by brief provider training, is therefore, a high‐priority, low‐cost intervention that could substantially accelerate screening and vaccination coverage.

A key strength of our study is its integration into an ongoing HOMINY prospective cohort midway through implementation, which enabled a rigorous, longitudinal mixed‐methods evaluation that few studies in the region have achieved. Embedding our intervention enabled the collection of detailed longitudinal quantitative data on socio‐demographic factors, CC screening and vaccination status using standardized, interviewer‐administered questionnaires to assess the impact. The qualitative component of the intervention sensitization on HPV knowledge, screening and vaccination provided a complementary lens to evaluate participants’ reactions, learning, behavioural change and outcomes.

Crucially, qualitative insights were strengthened by quantitative trends, allowing triangulation of findings and a nuanced understanding of the intervention's impact over time. The convergence of these data suggests that the observed changes reflect genuine community‐level behavioural shifts that translate awareness into action. Implementing FACT at this strategic midpoint enabled the team to measure pre‐sensitization knowledge and behaviours, deliver targeted education just before Nigeria's second‐phase national HPV vaccine rollout, and assess both immediate and longer‐term effects on HPV knowledge and vaccine awareness in participant dyads, uptake among adolescents, as well as HPV screening among mothers.

Several limitations temper these findings. First, all outcomes rely on self‐reported awareness and screening history, which may be affected by recall or social desirability bias. Second, the study may not be fully representative of all Nigerian communities, limiting generalizability to rural, primary care, or other geopolitical contexts. Third, some subgroup analyses, particularly stratifications by HIV status within the adolescent group, were underpowered. A much larger cohort across geopolitical zones in Nigeria will provide greater coverage and assessment. Fourth, and importantly, post‐sensitization data regarding source(s) of information that influenced uptake of HPV‐related services were not collected, making it difficult to distinguish the project's impact from the 5 days national vaccine drive despite the progressive increase in uptake over the 18‐month study period. Finally, the development and formal validation of HPV knowledge and attitude scales for SSA populations would strengthen future research and programme evaluation efforts.

To translate rising awareness into action, programmes should harness the channels that adolescents and their families already trust and use. Social media platforms and chatting apps popular with young people can host quizzes, short videos and personal testimonials that dispel myths and reduce stigma around HPV vaccination and screening. Training peer educators in schools and adolescent clubs to lead debates, run interactive sessions and share accurate information will further normalize preventive behaviours. At the same time, maternal influence can be reinforced by embedding HPV counselling and vaccination reminders into antenatal, postnatal and routine child‐immunization visits, so mothers receive clear guidance on their own screening needs and their children's eligibility for vaccination. Outreach should explicitly include boys, highlighting that HPV is also a cause of oropharyngeal and anogenital cancer and that they, too, are susceptible and need vaccination.

Parallel efforts must focus on expanding accessible screening and treatment services. Training and equipping midwives, nurses and community health workers to perform VIA and provide immediate treatment can rapidly extend coverage to underserved areas while gradually building laboratory capacity for HPV detection and cytology. This task‐shifting approach brings preventive care closer to households, reducing travel barriers and increasing early detection rates. Integrating these clinical services with the community‐driven education strategies creates a comprehensive, sustainable framework that protects women, men, boys and girls from HPV‐related cancers.

## 5 Conclusions

HPV vaccine awareness and uptake in this Nigerian cohort were critically low prior sensitization among adolescents but improved substantially following targeted community‐based programming aligned with research activities and active follow‐up of participants. VIA screening also increased significantly among mother−participants. These findings demonstrate that integrated, community‐led interventions embedded within HIV care and research platforms can effectively promote STI prevention and contribute to broader CC elimination goals. Healthcare provider recommendations emerged as a key determinant of screening behaviour. To stimulate action for the national HPV vaccination rollout, a combined strategy will be essential, one that includes strong community education to raise awareness, address misconceptions and vaccine mistrust, active engagement of adolescents and systematic prompts for healthcare providers. These components are critical to achieving the WHO's targets for eliminating cervical and other HPV‐related cancers. Additionally, improving access to HPV vaccination and scaling up screening among mothers will be vital for reducing the incidence of HPV‐associated cancers.

## Author Contributions

*Conceptualization*: MOC, PA, EO^2^, YB, NS, NO‐P, OO and FEE‐U. *Data curation*: MOC, AK, RA, PA and EO^2^. *Formal analysis*: EO, MOC and OP. *Funding acquisition*: MOC. *Investigation*: OP, EO^2^, PA and NLI. *Methodology*: OP, PA, EO^2^, NLI and MOC. *Project administration*: MOC, OO, FEE‐U, PA, EO^2^ and NLI. *Resources*: PA, EO^2^ and MOC. *Software*: MOC, PA, RA and AK. *Supervision*: MOC, PA and EO^2^. *Validation*: MOC, PA, EO^2^, OP and AK. *Visualization*: EO, MOC and OP. *Writing (original draft preparation)*: OP. *Writing (review and editing)*: OP, MOC, PA, EO^2^, POO‐A, NLI, EO and FEE‐U.

## Funding

The study was funded by NIH/NIDCR R01DE032216.

## Conflicts of Interest

The authors declare no conflicts of interest.

## Supporting information




**Supporting File 1**: Qualitative Discussion and Sensitisation Guide

**Supporting File 2**: Vaccine awareness and knowledge across visits (Stratification by gender and HIV acquisition)–Adolescents only

**Supporting File 3**: Vaccination uptake across visits (Stratification by gender and HIV acquisition)–Adolescents only

**Supporting File 4**: Sociodemographic Characteristics of Participants enrolled for the Qualitative data

## Data Availability

De‐identified datasets generated in this study are not publicly available but can be provided upon request from the corresponding author.

## References

[1] WHO , Human Papillomavirus (HPV) Vaccine Coverage Monitoring Manual (World Health Organization, 2020).

[2] H. Sung , J. Ferlay , and R. L. Siegel , “Global Cancer Statistics 2020: GLOBOCAN Estimates of Incidence and Mortality Worldwide for 36 Cancers in 185 Countries,” CA: A Cancer Journal for Clinicians 71 (2021): 209–249, 10.3322/caac.21660. 33538338

[3] M. L. Gillison , W. M. Koch , and R. B. Capone , “Evidence for a Causal Association Between Human Papillomavirus and a Subset of Head and Neck Cancers,” Journal of the National Cancer Institute 92 (2000): 709–720, 10.1093/jnci/92.9.709. 10793107

[4] IARC , “Human Papillomaviruses,” IARC Monographs on the Evaluation of Carcinogenic Risks to Humans 90 (2007): 1–177. 18354839 PMC4781057

[5] J. Ferlay , M. Ervik , and F. Lam , Statistics at a Glance (2022).

[6] B. K. Tadese , X. You , and T. Ndao , “The Burden of HPV Infections and HPV‐Related Diseases Among People With HIV: A Systematic Literature Review,” Journal of Medical Virology 97 (2025): e70274, 10.1002/jmv.70274. 40172095 PMC11963496

[7] G. M. Clifford , H. de Vuyst , V. Tenet , M. Plummer , S. Tully , and S. Franceschi , “Effect of HIV Infection on Human Papillomavirus Types Causing Invasive Cervical Cancer in Africa,” Journal of Acquired Immune Deficiency Syndromes 73 (2016): 332–339, 10.1097/QAI.0000000000001113. 27331659 PMC5172520

[8] K. S. Okunade , O. Sunmonu , G. E. Osanyin , and A. A. Oluwole , “Knowledge and Acceptability of Human Papillomavirus Vaccination Among Women Attending the Gynaecological Outpatient Clinics of a University Teaching Hospital in Lagos, Nigeria,” Journal of Tropical Medicine 2017 (2017): 8586459, 10.1155/2017/8586459. 29410683 PMC5749286

[9] M. C. Ezeanochie and B. N. Olagbuji , “Human Papilloma Virus Vaccine: Determinants of Acceptability by Mothers for Adolescents in Nigeria,” African Journal of Reproductive Health 18 (2014): 154–158. 25438520

[10] O. W. Akande and T. M. Akande , “Human Papillomavirus Vaccination Amongst Students in a Tertiary Institution in North Central Nigeria: A Cross‐Sectional Study on Sociodemographic Factors Associated With Its Awareness, Uptake and Willingness to Pay,” Nigerian Postgraduate Medical Journal 31 (2024): 14–24, 10.4103/npmj.npmj_265_23. 38321793

[11] D. Watson‐Jones , J. Changalucha , and H. Whitworth , “Immunogenicity and Safety of One‐Dose Human Papillomavirus Vaccine Compared With Two or Three Doses in Tanzanian Girls (DoRIS): An Open‐Label, Randomised, Non‐Inferiority Trial,” Lancet Global Health 10 (2022): e1473–e1484, 10.1016/S2214-109X(22)00309-6. 36113531 PMC9638030

[12] WHO , Nigeria to Vaccinate 7.7 Million Girls Against Leading Cause of Cervical Cancer (2023), https://www.afro.who.int/countries/nigeria/news/nigeria‐vaccinate‐77‐million‐girls‐against‐leading‐cause‐cervical‐cancer (accessed September 25, 2025).

[13] P. Adepoju , “Nigeria Targets Almost 8 Million Girls With HPV Vaccine,” Lancet 402 (2023): 1612, 10.1016/S0140-6736(23)02450-9. 37926106

[14] R. V. Barnabas , E. R. Brown , and M. A. Onono , “Durability of Single‐Dose HPV Vaccination in Young Kenyan Women: Randomized Controlled Trial 3‐Year Results,” Nature Medicine 29 (2023): 3224–3232, 10.1038/s41591-023-02658-0. PMC10719107 38049621

[15] K. E. Gallagher , D. S. LaMontagne , and D. Watson‐Jones , “Status of HPV Vaccine Introduction and Barriers to Country Uptake,” Vaccine 36 (2018): 4761–4767, 10.1016/j.vaccine.2018.02.003. 29580641

[16] WHO , Global Strategy to Accelerate the Elimination of Cervical Cancer as a Public Health Problem (World Health Organization, 2020).

[17] M. A. Habila , L. J. Kimaru , and N. Mantina , “Community‐Engaged Approaches to Cervical Cancer Prevention and Control in Sub‐Saharan Africa: A Scoping Review,” Frontiers in Global Women's Health 2 (2021): 697607, 10.3389/FGWH.2021.697607/FULL. PMC8594022 34816234

[18] M. O. Coker , N. F. Schlecht , and E. Osagie , “Baseline Prevalence of Oral Human Papillomavirus in Mother−Child Pairs With and Without HIV Infection,” JAMA Network Open 7 (2024): e2451512, 10.1001/jamanetworkopen.2024.51512. 39688870 PMC11653123

[19] A. Akinyemi , A. Akintokun , and A. E. Knowledge , “Attitude, and Preventive Practices on Human Papillomavirus Vaccination Among Mothers of Adolescent Girls in Selected Secondary Schools of Lagos, Nigeria,” European Medical Journal 9 (2024): 102–112, 10.33590/emj/GMJC4983.

[20] J. I. Rosser , B. Njoroge , and M. J. Huchko , “Changing Knowledge, Attitudes, and Behaviors Regarding Cervical Cancer Screening: The Effects of an Educational Intervention in Rural Kenya,” Patient Education and Counseling 98 (2015): 884–889, 10.1016/J.PEC.2015.03.017. 25858634 PMC4437717

[21] M. O. Ogbolu and M. Kozlovszky , “Assessment of HPV Knowledge and Awareness Among Students and Staff at IBB University, Niger State, Nigeria: Implications for Health Education and Prevention,” Healthcare (Basel) 12, no. 6 (2024): 665, 10.3390/HEALTHCARE12060665. 38540629 PMC10970435

[22] E. Osagie , P. Akhigbe , and N. Idemudia , “Human Papillomavirus, Human Immunodeficiency Virus, and Oral Microbiota Interplay in Nigerian Youth (HOMINY): A Prospective Cohort Study Protocol,” BMJ Open 15 (2025): e091017, 10.1136/bmjopen-2024-091017. PMC11808902 39922591

[23] G. Dubourg , M. Surenaud , Y. Lévy , S. Hüe , and D. Raoult , “Microbiome of HIV‐Infected People,” Microbial Pathogenesis 106 (2017): 85–93, 10.1016/j.micpath.2016.05.015. 27216237

[24] UNICEF , Nigeria to Vaccinate 7.7 Million Girls Against Leading Cause of Cervical Cancer (2023).

[25] D. L. Kirkpatrick and J. D. Kirkpatrick , Evaluating Training Programs: The Four Levels (Berrett‐Koehler Publishers, 2006).

[26] M. Ezeanochie and P. Olasimbo , “Awareness and Uptake of Human Papilloma Virus Vaccines Among Female Secondary School Students in Benin City, Nigeria,” African Health Sciences 20 (2020): 45–50, 10.4314/ahs.v20i1.8. 33402891 PMC7750089

[27] H. Fisher , S. Denford , S. Audrey , et al., “Information Needs of Ethnically Diverse, Vaccine‐Hesitant Parents During Decision‐Making About the HPV Vaccine for Their Adolescent Child: A Qualitative Study,” BMC Public Health 24, no. 1 (2024): 91, 10.1186/s12889-023-17540-4. 38178083 PMC10768213

[28] J. Waller , R. Ostini , L. A. V. Marlow , K. McCaffery , and G. Zimet , “Validation of a Measure of Knowledge About Human Papillomavirus (HPV) Using Item Response Theory and Classical Test Theory,” Preventive Medicine (Baltimore) 56 (2013): 35–40, 10.1016/j.ypmed.2012.10.028. 23142106

[29] L. A. Palinkas , S. M. Horwitz , C. A. Green , J. P. Wisdom , N. Duan , and K. Hoagwood , “Purposeful Sampling for Qualitative Data Collection and Analysis in Mixed Method Implementation Research,” Administration and Policy in Mental Health and Mental Health Services Research 42 (2015): 533–544, 10.1007/s10488-013-0528-y. 24193818 PMC4012002

[30] P. A. Harris , R. Taylor , and B. L. Minor , “The REDCap Consortium: Building an International Community of Software Platform Partners,” Journal of Biomedical Informatics 95 (2019): 103208, 10.1016/j.jbi.2019.103208. 31078660 PMC7254481

[31] R Core Team , R: A Language and Environment for Statistical Computing (2025).

[32] S. Perlman , R. G. Wamai , P. A. Bain , T. Welty , E. Welty , and J. G. Ogembo , “Knowledge and Awareness of HPV Vaccine and Acceptability to Vaccinate in Sub‐Saharan Africa: A Systematic Review,” PLoS ONE 9 (2014): e90912, 10.1371/JOURNAL.PONE.0090912. 24618636 PMC3949716

[33] J. Musa , C. J. Achenbach , and L. C. O'Dwyer , “Effect of Cervical Cancer Education and Provider Recommendation for Screening on Screening Rates: A Systematic Review and Meta‐Analysis,” PLoS ONE 12 (2017): e0183924, 10.1371/JOURNAL.PONE.0183924. 28873092 PMC5584806

[34] T. Rana , D. N. S. Chan , B. M. H. Law , K. C. Choi , S. Shrestha , and W. K. W. So , “Determinants of Cervical Cancer Screening Utilisation Among Women in the Least Developed Countries: A Systematic Review and Meta‐Analysis,” PLOS One 20, no. 6 (2025): e0321627. 10.1371/journal.pone.0321627. 40554550 PMC12186883

[35] E. Kwigizile , E. Shao , G. Mtango , T. Sonda , J. Moshi , and J. Chilongola , “The Gap Between Knowledge and Practice of Risky Sexual Behaviors for HIV Among University Students and Staff in Moshi Town in Tanzania,” Journal of Public Health in Africa 4 (2013): e8, 10.4081/jphia.2013.e8. 28299097 PMC5345427

[36] I. M. Oharume , “Knowledge, Sexual Behaviours and Risk Perception of Sexually Transmitted Infections Among Students of the Polytechnic, Ibadan, Oyo State,” African Health Sciences 20 (2020): 39–44, 10.4314/ahs.v20i1.7. 33402890 PMC7750043

[37] L. I. Abugu and E. N. Nwagu , “Awareness, Knowledge and Screening for Cervical Cancer Among Women of a Faith‐Based Organization in Nigeria,” Pan African Medical Journal 39 (2021), 10.11604/pamj.2021.39.200.23761. PMC8464205 34603581

[38] A. A. Okunowo , A. O. Ugwu , J. O. Kuku , et al., “Predictors, Barriers and Motivating Factors for Human Papillomavirus Vaccination and Testing as Preventive Measures for Cervical Cancer: A Study of Urban Women in Lagos, Nigeria,” Preventive Medicine Reports 24 (2021): 101643, 10.1016/j.pmedr.2021.101643. 34987955 PMC8693866

[39] F. M. Balogun and O. O. Omotade , “Parental Intention to Vaccinate Adolescents With HPV Vaccine in Selected Communities in Ibadan, Southwest Nigeria: An Application of Integrated Behavioral Model,” Human Vaccines & Immunotherapeutics 18no. 5 (2022): 2069959, 10.1080/21645515.2022.2069959. 35561294 PMC9359392

[40] J. M. Kutz , P. Rausche , T. Gheit , D. I. Puradiredja , and D. Fusco , “Barriers and Facilitators of HPV Vaccination in Sub‐Saharan Africa: A Systematic Review,” BMC Public Health [Electronic Resource] 23 (2023), 10.1186/s12889-023-15842-1. PMC10214362 37237329

[41] K. Unfried and J. Priebe , “Vaccine Hesitancy and Trust in Sub‐Saharan Africa,” Scientific Reports 14, no. 1 (2024): 10860, 10.1038/s41598-024-61205-0. 38740790 PMC11091197

