# Supplementary material for: From Awareness to Action Study: Improving Human Papillomavirus Knowledge, Screening and Vaccine Uptake Among Mother‐Adolescent Pairs in the HOMINY Study in Nigeria: A Longitudinal Study
Source: J Int AIDS Soc. 2026 Jul 24;29(Suppl 2):e70164. doi: 10.1002/jia2.70164 (PMC13400979; doi:10.1002/jia2.70164)
Supplement: Supplementary file 1 — Supporting File 1: Qualitative Discussion and Sensitisation Guide [file JIA2-29-e70164-s002.docx]

**Supplementary File 1:** **Qualitative Discussion and Sensitisation Guide**

**Qualitative Discussion Questions**

The qualitative discussions explored participants’ understanding and perceptions of Human Papillomavirus (HPV) infection, transmission modes, cervical cancer, cervical cancer screening, and HPV vaccination. Discussions lasted approximately 60-90 minutes.

Key discussion questions included:

1. What do you know about HPV infection?
2. What causes HPV infection?
3. How is HPV transmitted?
4. Are there signs or symptoms of HPV infection?
5. What have you heard about cervical cancer?
6. Have you heard about the HPV vaccine?
7. Who can receive the HPV vaccine?
8. Where can the HPV vaccine be received?
9. Can the HPV vaccine prevent cervical cancer?
10. What does it cost to screen for cervical cancer?

**Summary of Sensitisation Content**

Participants received educational information on:

- Definition and overview of Human Papillomavirus (HPV)
- Modes of HPV transmission
- Cervical cancer prevention strategies
- HPV vaccination eligibility and schedule
- Cervical cancer screening eligibility and methods
- Importance of early detection and prevention

The sensitisation sessions were designed to improve participants’ understanding of HPV infection, cervical cancer prevention, and participation throughout the study.

**Questionnaire Domains and Assessment Prompts**

**HPV Knowledge**

Participants were asked about:

- Awareness of HPV
- Knowledge of the association between HPV and cancer
- Knowledge of HPV transmission routes (sexual contact, kissing, and mother-to-child transmission)
- Knowledge of HPV prevention through vaccination

**HPV Screening**

Participants were asked about:

- Uptake of HPV screening since the previous study visit (Visual Inspection with Acetic Acid or Papanicolaou test)
- Reasons for non-participation in HPV screening among those who had not undergone screening

**HPV Vaccination**

Participants were asked about:

- Awareness of the HPV vaccine since the previous study visit
- Receipt of any HPV vaccine dose since the previous study visit
