# Supplementary material for: From Awareness to Action Study: Improving Human Papillomavirus Knowledge, Screening and Vaccine Uptake Among Mother‐Adolescent Pairs in the HOMINY Study in Nigeria: A Longitudinal Study
Source: J Int AIDS Soc. 2026 Jul 24;29(Suppl 2):e70164. doi: 10.1002/jia2.70164 (PMC13400979; doi:10.1002/jia2.70164)
Supplement: Supplementary file 2 — Supporting File 2: Vaccine awareness and knowledge across visits (Stratification by gender and HIV acquisition)–Adolescents only [file JIA2-29-e70164-s001.docx]

**Supplementary File 2: Vaccine awareness and knowledge across visits (Stratification by gender and HIV acquisition) – Adolescents only**


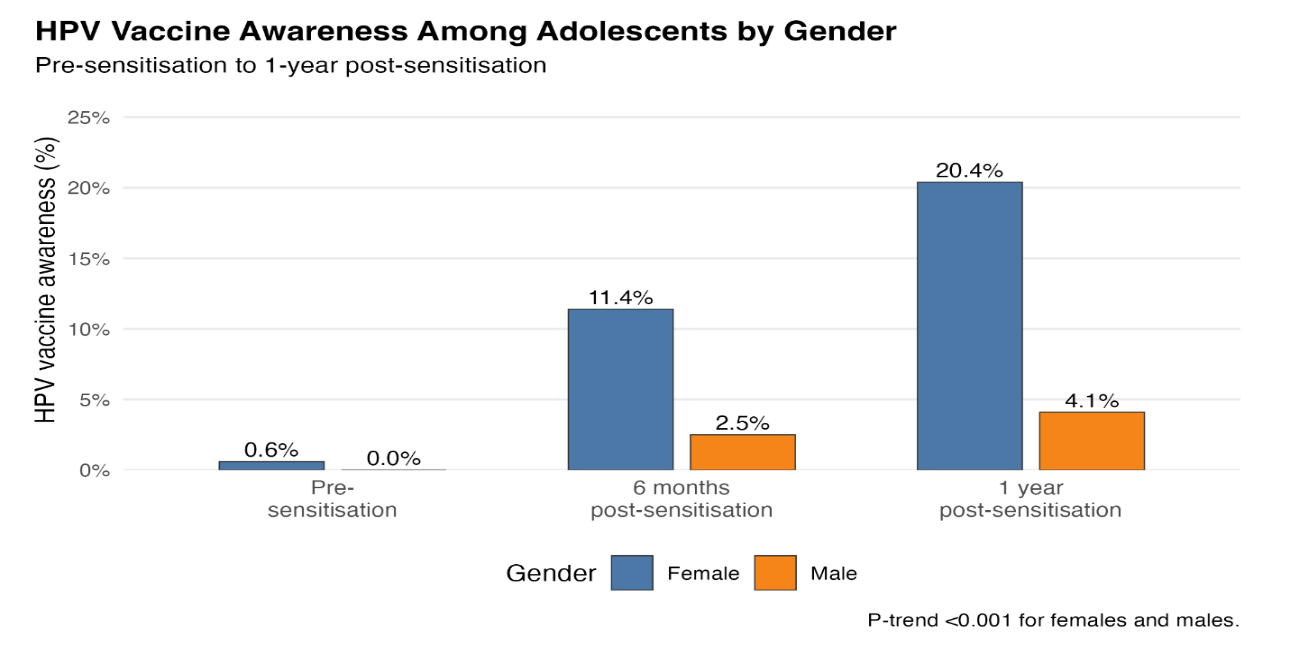


Figure 1. **Shows HPV vaccine awareness by gender of adolescents enrolled in the FACT study.**


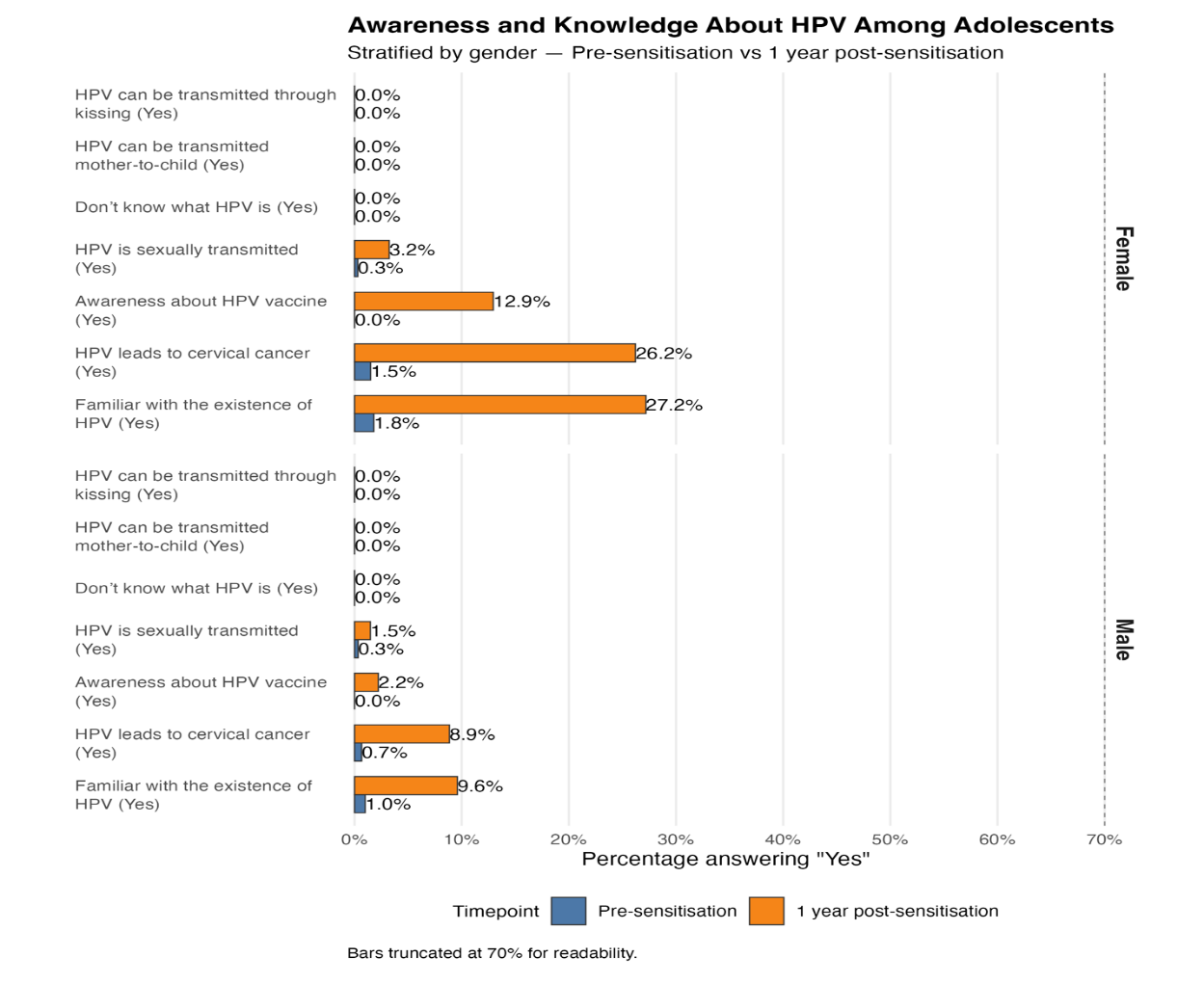


Figure 2**. HPV vaccine knowledge improvements of FACT study participants across the timepoints stratified by gender.**
