# Supplementary material for: From Awareness to Action Study: Improving Human Papillomavirus Knowledge, Screening and Vaccine Uptake Among Mother‐Adolescent Pairs in the HOMINY Study in Nigeria: A Longitudinal Study
Source: J Int AIDS Soc. 2026 Jul 24;29(Suppl 2):e70164. doi: 10.1002/jia2.70164 (PMC13400979; doi:10.1002/jia2.70164)
Supplement: Supplementary file 3 — Supporting File 3: Vaccination uptake across visits (Stratification by gender and HIV acquisition)–Adolescents only [file JIA2-29-e70164-s004.docx]

**Supplementary File 3: Vaccination uptake across visits (Stratification by gender and HIV acquisition) – Adolescents only**

**Table 1** shows changes in HPV vaccine uptake by gender.

| Gender | Visit | N | HPV vaccine dose 1 (%) | Dose p values |
| --- | --- | --- | --- | --- |
| Female | Pre-sensitisation | 334 | 0 (0) | < 0.0001 |
| Female | 6-month visit | 317 | 14 (4.4%) | < 0.0001 |
| Female | 1-year visit | 309 | 20 (6.5%) | < 0.0001 |
| Male | Pre-sensitisation | 302 | 0 (0%) |  |
| Male | 6-month visit | 280 | 0 (0%) |  |
| Male | 1-year visit | 271 | 0 (0%) |  |

**Table 2** shows HIV acquisition status from pre-sensitisation to 1-year post-sensitisation.

| **Study group** | **Visit** | **N** | **HPV vaccine dose 1 (%)** | **Dose p values** |
| --- | --- | --- | --- | --- |
| HI | Pre-sensitisation | 220 | 0 (0) | 0.003 |
| HI | 6-month visit | 209 | 4 (1.9%) | 0.003 |
| HI | 1-year visit | 199 | 8 (4%) | 0.003 |
| HEU | Pre-sensitisation | 203 | 0 (0%) | <0.001 |
| HEU | 6-month visit | 196 | 3 (1.5%) | <0.001 |
| HEU | 1-year visit | 192 | 9 (4.7%) | <0.001 |
| HUU | Pre-sensitisation | 213 | 0 (0%) | 0.186 |
| HUU | 6-month visit | 192 | 7 (3.6%) | 0.186 |
| HUU | 1-year visit | 189 | 3 (1.6%) | 0.186 |
| **Study group codes: HI** = adolescents living with HIV; **HEU** = adolescents HIV-exposed without acquisition; **HUU** = adolescents HIV-unexposed. | | | | |


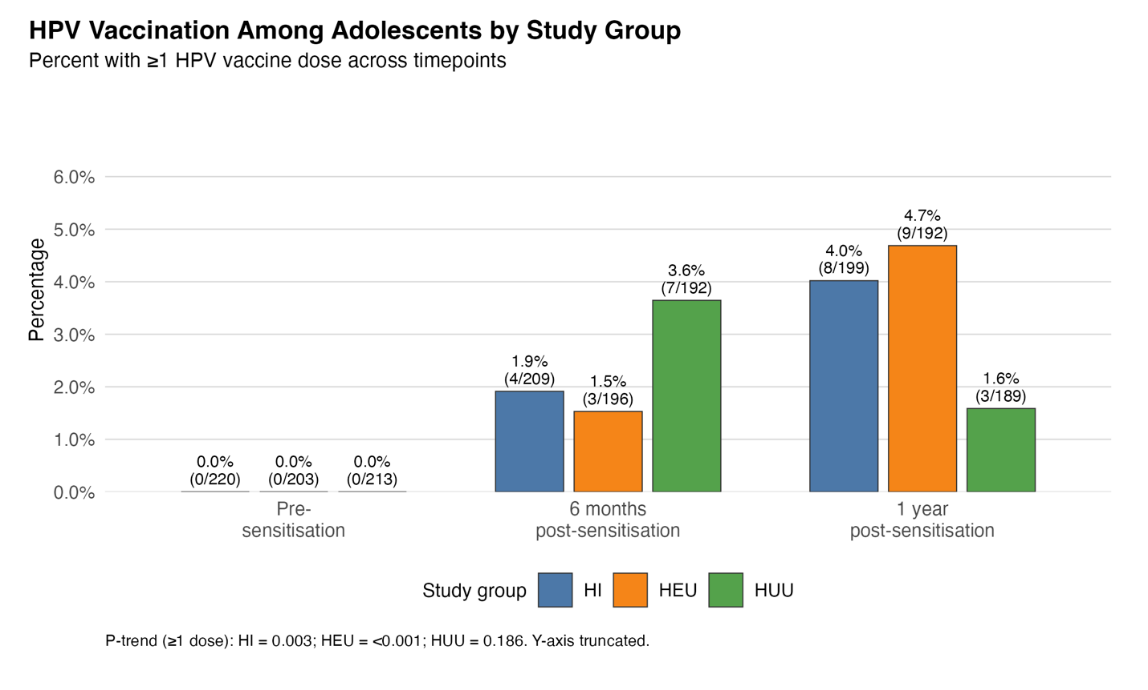


Figure 1**. Shows the trend of HPV vaccine uptake by HIV acquisition.**

**Study group codes: HI** = adolescents living with HIV; **HEU** = adolescents HIV-exposed without acquisition; **HUU** = adolescents HIV-unexposed.
