# Supplementary material for: From Awareness to Action Study: Improving Human Papillomavirus Knowledge, Screening and Vaccine Uptake Among Mother‐Adolescent Pairs in the HOMINY Study in Nigeria: A Longitudinal Study
Source: J Int AIDS Soc. 2026 Jul 24;29(Suppl 2):e70164. doi: 10.1002/jia2.70164 (PMC13400979; doi:10.1002/jia2.70164)
Supplement: Supplementary file 4 — Supporting File 4: Sociodemographic Characteristics of Participants enrolled for the Qualitative data [file JIA2-29-e70164-s003.docx]

## Supplementary File 4: Sociodemographic Characteristics of Participants enrolled for the Qualitative data

## Table 1. Mother characteristics

| **Characteristics** | **Overall**  N = 87^1^ | **HI**  N = 38^1^ | **HU**  N = 49^1^ | **p-value**^2^ |
| --- | --- | --- | --- | --- |
| **Age, years** | 41.0 (5.5) | 41.7 (5.4) | 40.4 (5.6) | 0.3 |
| **Highest level of education** |  |  |  | <0.001 |
| Primary | 13 (15%) | 10 (27%) | 3 (6.1%) |  |
| JSS | 8 (9.3%) | 2 (5.4%) | 6 (12%) |  |
| SSS | 43 (50%) | 23 (62%) | 20 (41%) |  |
| Certificate/Diploma | 11 (13%) | 0 (0%) | 11 (22%) |  |
| University degree | 8 (9.3%) | 0 (0%) | 8 (16%) |  |
| Other | 3 (3.5%) | 2 (5.4%) | 1 (2.0%) |  |
| Missing | 1 | 1 | 0 |  |
| **Monthly income, Naira** | 53,386 (55,351) | 43,222 (48,580) | 61,170 (59,355) | 0.032 |
| Missing | 4 | 2 | 2 |  |
| **Earns wages or income** |  |  |  | >0.9 |
| Yes | 83 (100%) | 36 (100%) | 47 (100%) |  |
| Missing | 4 | 2 | 2 |  |
| **Current employment status** |  |  |  | 0.013 |
| Professional | 6 (6.9%) | 0 (0%) | 6 (12%) |  |
| Clerical | 9 (10%) | 1 (2.6%) | 8 (16%) |  |
| Skilled manual | 12 (14%) | 7 (18%) | 5 (10%) |  |
| Unskilled work | 56 (64%) | 27 (71%) | 29 (59%) |  |
| Unemployed | 4 (4.6%) | 3 (7.9%) | 1 (2.0%) |  |
| **Current viral load category** |  |  |  | >0.9 |
| <20 copies/mL | 30 (79%) | 30 (79%) | 0 (NA%) |  |
| 20+ copies/mL | 8 (21%) | 8 (21%) | 0 (NA%) |  |
| Missing | 49 | 0 | 49 |  |
| **Current viral load value, copies/mL** | 33,727 (83,749) | 33,727 (83,749) | NA (NA) |  |
| Missing | 77 | 28 | 49 |  |
| ^1^Mean (SD); n (%)  ^2^Wilcoxon rank sum test; Fisher's exact test; NA  **SD:** standard deviation; **Study group codes: HI** = mothers living with HIV; **HU** = mothers HIV-unexposed. | | | | |

## Table 2. Youth characteristics

| **Characteristics** | **Overall**  N = 93^1^ | **HI**  N = 42^1^ | **HUU**  N = 51^1^ | **p-value**^2^ |
| --- | --- | --- | --- | --- |
| **Sex** |  |  |  | 0.7 |
| Male | 33 (35%) | 14 (33%) | 19 (37%) |  |
| Female | 60 (65%) | 28 (67%) | 32 (63%) |  |
| **Age, years** | 10.46 (0.94) | 10.52 (0.83) | 10.41 (1.02) | 0.5 |
| **Highest level of education** |  |  |  | 0.083 |
| Primary | 62 (67%) | 32 (76%) | 30 (59%) |  |
| JSS | 31 (33%) | 10 (24%) | 21 (41%) |  |
| **Currently enrolled in school** | 93 (100%) | 42 (100%) | 51 (100%) | >0.9 |
| **Type of school attended** |  |  |  | 0.8 |
| Private school | 67 (74%) | 29 (73%) | 38 (76%) |  |
| Public school | 23 (26%) | 11 (28%) | 12 (24%) |  |
| Missing | 3 | 2 | 1 |  |
| **School type history** |  |  |  | 0.6 |
| Same type of school | 68 (76%) | 29 (73%) | 39 (78%) |  |
| Switched type of school | 22 (24%) | 11 (28%) | 11 (22%) |  |
| Missing | 3 | 2 | 1 |  |
| **Additional lessons or after-school teaching** |  |  |  | 0.047 |
| Yes, currently | 58 (64%) | 26 (65%) | 32 (64%) |  |
| Yes, in the past | 25 (28%) | 8 (20%) | 17 (34%) |  |
| No | 7 (7.8%) | 6 (15%) | 1 (2.0%) |  |
| Missing | 3 | 2 | 1 |  |
| ^1^n (%); Mean (SD)  ^2^Pearson's Chi-squared test; Wilcoxon rank sum test; Fisher's exact test  **SD:** standard deviation; **Study group codes: HI** = adolescents living with HIV; **HEU** = adolescents HIV-exposed without acquisition; **HUU** = adolescents HIV-unexposed. | | | | |
